# Effect of Methylphenidate in Patients with Cancer-Related Fatigue: A Systematic Review and Meta-Analysis

**DOI:** 10.1371/journal.pone.0084391

**Published:** 2014-01-08

**Authors:** Shun Gong, Ping Sheng, Hai Jin, Hua He, Enbo Qi, Wen Chen, Yan Dong, Lijun Hou

**Affiliations:** 1 Department of Neurosurgery, Shanghai Institute of Neurosurgery, PLA Institute of Neurosurgery, Changzheng Hospital, Second Military Medical University, Shanghai, China; 2 Neuroscience Center, Changzheng Hospital, Second Military Medical University, Shanghai, China; University of Louisville, United States of America

## Abstract

**Background:**

Cancer-related fatigue (CRF) is a common symptom affecting patients with cancer. There are an increasing number of trials examining potential treatments for CRF. Methylphenidate represents one of the most researched drugs and an up-to-date assessment of the evidence for its use is needed. Trials of methylphenidate for CRF provided inconsistent results. This meta-analysis was aimed at assessing the effect and safety of methylphenidate on CRF.

**Methods:**

We comprehensively searched the Pubmed, EMBASE, PSYCHInfo and the Cochrane databases in order to identify published studies on the effect of methylphenidate on CRF. Primary outcomes included fatigue. Secondary outcomes included depression, cognition and adverse effects.

**Findings:**

A meta-analysis was conducted on five randomized controlled trials and 498 patients were enrolled. Despite a large placebo effect observed in the studies included, pooled data suggested therapeutic effect of methylphenidate on CRF. Subgroup Analyses showed that the efficacy of methylphenidate on CRF is getting better with prolonging treatment duration, with a MD of −3.70 (95% CI −7.03– −0.37, p = 0.03) for long-time group and a MD of −2.49 (95% CI −6.01–1.03, p = 0.17) for short-time group. In general, there was no impact of methylphenidate on depression and cognition associated with CRF. Adverse events were similar between methylphenidate and placebo groups except that more patients reported vertigo, anxiety, anorexia and nausea in methylphenidate group compared to placebo group.

**Conclusion:**

Existing trials of methylphenidate on CRF provided limited evidence for the use of methylphenidate to treat CRF. The absolute numbers still remain small, and further confirmation is needed before firm recommendations on their usage and safety can be made in the treatment of CRF.

## Introduction

Cancer-related fatigue (CRF) is a significant clinical problem affecting patients at all stages of treatment and increases with advanced diseases [1]. CRF is defined as “a distressing persistent, subjective sense of tiredness or exhaustion related to cancer or cancer treatment that is not proportional to recent activity and interferes with usual functioning” [2]. 60% to 90% of patients with advanced cancer declare CRF as the most frequent and debilitating symptom interfering with a patient's ability to perform physical tasks and participate in social activities [3]–[5]. The patients feel that it imposes a larger impact on their daily lives than pain, depression, or nausea [6]. At present, there is no clearly superior treatment for CRF. Management options include the use of exercise and psychosocial interventions [7]–[8]. For some patients, pharmacological interventions consisting of prescription of low-dose steroids, modafinil, and psychostimulants, such as methylphenidate, dexamphetamine or pemoline may be appropriate [9]. Among these modalities that have been evaluated to date, methylphenidate seems to be the most promising pharmacological agent for CRF.

Methylphenidate is a psychostimulant with its main application in the treatment of attention deficit disorder (ADD) [10], which acts to increase the levels of dopamine in the central nervous system [11]. Methylphenidate has been used beyond license for various indications in patients with advanced diseases, i.e. in opioid-induced sedation, in the treatment of depression, and in the management of fatigue [12]–[14]. Many earlier studies point to it as an effective treatment that is well tolerated in patients with various types of cancer [15]–[24]. But the evidence for the efficacy of methylphenidate in the setting of CRF is weak, mainly extrapolated from randomized studies in other diseases or other symptoms, or based on non-randomized trials. For instance, both Johnson et al. and Gehring et al. provided support for the use of methylphenidate to treat fatigue [23]–[24], with several limitations including the small number of patients, limited follow-up time, open label design and lack of placebo. Other studies showed the effectiveness of methylphenidate mostly came from experience treatment, self-control and other drugs control. As they ignored the effect of placebo, further studies are needed to quantify the placebo effect.

Recently there have been several control studies and meta-analyses reported investigating the impact of methylphenidate on CRF [25]–[33]. However, these trials showed inconsistent results. For instance, both Butler et al. and Bruera et al. failed to demonstrate any statistically significant benefit of methylphenidate over placebo [28]–[29]. On the contrary, Cueva et al. showed the effectiveness of methylphenidate in attenuating asthenia in breast carcinoma patients who received chemotherapy [30]. Clinical characteristics are a good predictor of ultimate and long-term response to methylphenidate therapy [34]. Hence, there is need to understand whether specific patient characteristics or other factors are associated with response to methylphenidate used for the treatment of CRF.

The purpose of this study is to specifically focus on increasing evidence for the use of methylphenidate in the treatment of CRF and to assess the efficacy and safety of methylphenidate in the treatment of CRF that will enable us to personalize the use of methylphenidate to only the patients who respond to this treatment.

## Methods

### Selection of Studies

The overview of RCTs was conducted in accordance with the Preferred Reporting Items for Systematic Reviews and Meta-analysis (PRISMA) statement [35]. We systematically searched PubMed, Embase, PSYCHInfo, and the Cochrane Library from their inception until the first week of June 2013 without language restriction, and identified all RCTs related to the effects of methylphenidate in patients with CRF. We used the following search keywords: “methylphenidate”, “dexmethylphenidate”, “d-MPH”, “ritalin”, “cancer”, “tumor”, “carcinoma”, “neoplasms”, “fatigue”, “asthenia”, “tiredness”, “CRF” and “randomized controlled trial”. Additionally, we manually searched the references of selective papers to identify additional potentially eligible studies.

### Inclusion criteria

Original studies were considered for inclusion in the meta-analysis if they met with the following criteria: (1) they were randomized controlled trials (RCT); (2) patients over 18 years old with cancer such as Breast, Prostate, Lung, Genitourinary, Gastrointestinal, Hematologic and Brain tumor were investigated; (3) the efficacy of methylphenidate on fatigue were examined; (4) results were sufficient to allow calculation of effect sizes.

### Data extraction and quality assessment

Two assessors (SG and PS) independently reviewed the full manuscripts of eligible studies. Data were extracted independently in standardized data-collection forms. Extracted data included first author's name, year of publication, sample size, patients' characteristics (mean age, gender), type of cancer, dosage of treatment, duration of treatment, outcomes, study design and country. Any discrepancy was resolved by discussion or a third author (YD). Selected RCTs were critically appraised using the Jadad scale, which scores studies' description of randomization (2 points), blinding (2 points) and attrition information (1 point) [36].

### Study Outcomes

The primary outcomes included fatigue scores measured by the Functional Assessment of Cancer Therapy-Fatigue subscale (FACT-F) [37] and the Brief Fatigue Inventory (BFI) [38] scores. The secondary outcomes included the depression, cognition and adverse effects. The subgroup analysis was performed based on duration of treatment.

### Statistical Analysis

For dichotomous data, the impact of the intervention was expressed as relative risk (RR) with 95% confidence intervals (CI) using the Mantel-Haenszel method. For continuous data the difference in change from baseline to follow-up between intervention and control groups was expressed as mean differences with 95% CI (if the same scale was used in all studies) or standardized mean differences with 95% CI (when different scales were used) using inverse variance method. Heterogeneity of treatment effects between studies was statistically explored by the I^2^ statistic. I^2^ statistic of 0%–40% indicates unimportant heterogeneity, 30%–60% indicates moderate heterogeneity, 50%–90% indicates substantial heterogeneity, and 75%–100% indicates considerable heterogeneity [39]. All reported P values were two-sides and P values less than 0.05 were deemed as statistically significant.

## Results

### Study characteristics

**Figure 1:**
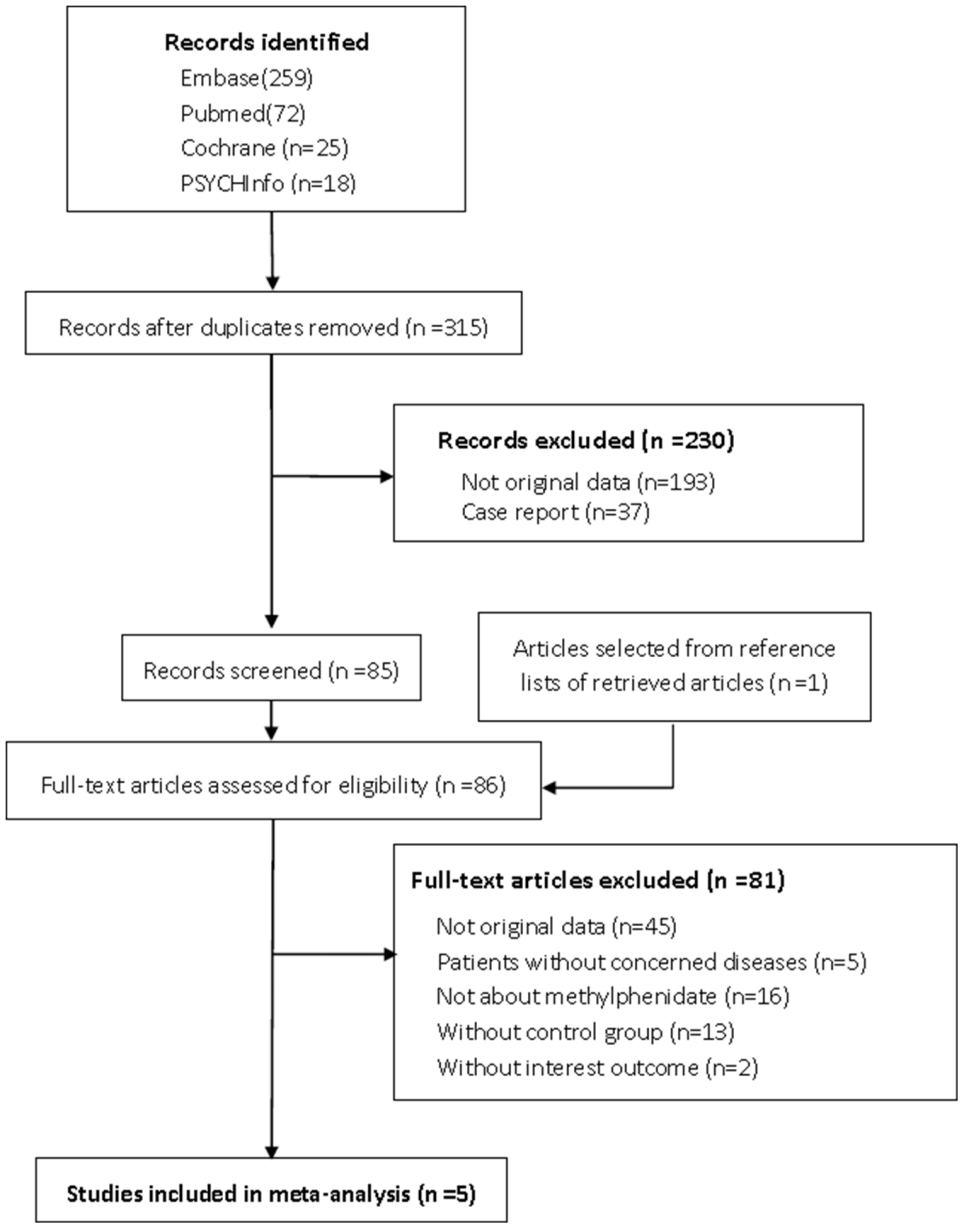
The flowchart shows the selection of studies for meta-analysis.

A total of 315 citations were identified from the electronic searches and 1 through other sources, of which 230 were excluded after a preliminary review. The remaining 86 studies were retrieved for detailed assessment. Ultimately, 5 RCTs met the inclusion criteria (Figure 1). All studies were of good quality with a score of 3 or more assessed by Jadad scale. Of 5 studies, 3 were identified in mixed tumor, 1 in primary brain tumor and 1 in prostate cancer. Two studies were conducted in multiple centers in one country and the other 3 at a single center. All studies were double blind and parallel design. The included studies consist of 498 patients (Table 1).

**Table 1:** Design and patient characteristics for studies included in the meta analysis.

source	Sample size methylphenidate placebo		sex (male)	mean age (year)	duration of treatment (week)	maximum dosage (mg/d)	type of cancer	Outcomes	baseline findings (mean ± SD, Methylphenidate/ placebo)	country	study design	Jadad score
Bruera et al, 2006	52	53	37%	57	1	20	Mixed tumor	Fatigue	FACT-F: 16.87±7.96/17.04±7.98	USA	parallel	5
								Depression	ESAS-D: 3.4±2.9 *			
Butler et al, 2007	20	21	54%	56	4	30	Primary brain tumor	Fatigue	FACT-F: 34.7±8.04/33.3±12.92	USA	parallel	4
								Depression	CESD: 14.6±8.62*			
								Cognition	MMSE: 27.2±2.92/25.6±3.39			
Lower et al, 2009	54	69	10%	53	8	28	Mixed tumor	Fatigue	FACT-F: 30.9±10.2/30.0±10.1	USA	parallel	5
								Depression	BDI-II:10.8±4.6/10.9±4.6			
								Cognition	MMSE: 28.7±1.7/28.8±1.5			
									HSCS:35.9±17.0/37.1±18.1			
Moraska et al, 2010	62	63	40%	60	4	54	Mixed tumor	Fatigue	BFI: 3.36±1.54/3.4±1.72	USA	parallel	5
Roth et al, 2010	10	13	100%	70	6	30	Prostate cancer	Fatigue	BFI: 5.13±2.25/4.01±2. 00	USA	parallel	3
									FSS: 4.27±1.31/4.21±1.37			

For both methylphenidate and placebo groups.

Abbreviations: FACT-F =  Functional Assessment of Cancer Therapy-Fatigue subscale; ESAS-D =  Edmonton Symptom Assessment System-Depression subscale; CESD =  Center for Epidemiologic Studies Depression Scale; MMSE =  Mini-Mental State Exam; BDI-II = Beck Depression Inventory-II; HSCS =  High Sensitivity Cognitive Screen; BFI =  Brief Fatigue Inventory; FSS = Fatigue Severity Scale.

### Systematic review of literature

The study by Bruera et al. examined the effect of methylphenidate on CRF in patients with advanced cancer [29]. Mixed types of tumors were included and the largest single group was breast cancer. Methylphenidate 5 mg or matching placebo was given on an ‘‘as needed’’ basis initiated by the patients themselves over a one-week period. The dose could be increased up to 20 mg per day by the patient, depending on response. In both groups, there was an improvement in fatigue scores measured by the FACT-F. However, no statistically significant difference was found between methylphenidate and placebo group on day 8.

Patients undergoing cranial radiotherapy with either primary or metastatic brain tumors were entered into the study of Butler et al. [28]. The dose was initiated with methylphenidate 5 mg twice daily or matching placebo and was increased to a maximum of methylphenidate 15 mg twice daily. The primary outcome was the change in fatigue score measured by the FACT-F at eight weeks after the completion of radiotherapy. There was, however, a high dropout rate over time, and the final analysis was conducted on a smaller sample size than the original one. A number of time points were examined and the fluctuation in fatigue scores was observed. However, no fatigue scores between treatment and placebo groups were significantly different at any time points.

Lower et al. have undertaken a randomized, double-blind study evaluated the potential therapeutic effect and safety of methylphenidate in the treatment of patients with chemotherapy-related fatigue [31]. The tumor groups studied were predominantly breast and ovarian and the exact distribution of disease stages was not described in the original article. Patients were randomized to methylphenidate given initially at 5 mg twice daily, increaed to a maximum of 50 mg per day over an eight-week period or to identically matched placebo tablet. Compared with placebo, methylphenidate-treated subjects showed a significant improvement in fatigue symptoms in the FACIT-F at eight weeks. An exploratory analysis also demonstrated significant differences in the FACT-F scores at a number of other time points.

Moraska et al. reported the impact of methylphenidate on cancer-related fatigue with interesting data [32]. In their study, patients with a history of cancer-related fatigue were included. Participants took one tablet on days 1 through 7, two tablets on days 8 through 14, and three tablets on days 15 through 28. Each methylphenidate tablet was 18 mg, resulting in the goal dose of 54 mg per day for the final 2 weeks of the study. The primary end point of the BFI did not show a statistically significant difference between the methylphenidate and placebo arms. However, a subset analysis suggested that patients with more severe fatigue and/or with more advanced disease did have some fatigue improvement with methylphenidate.

The efficacy of methylphenidate on CRF was examined by Roth et al. in patients with advanced prostate cancer and the presence of moderate to severe fatigue [33]. Methylphenidate 5 mg or matching placebo was given on an ‘‘as needed’’ basis initiated by the patients themselves over a six-week period. The dose could be increased up to 30 mg per day by the patient, depending on response. Compared with placebo group, the methylphenidate group reported greater decrease on BFI severity scores (*p* = 0.03) and a trend toward greater decrease on BFI total scores (*p* = 0.03).

### Efficacy of methylphenidate on Cancer-related fatigue

**Figure 2:**
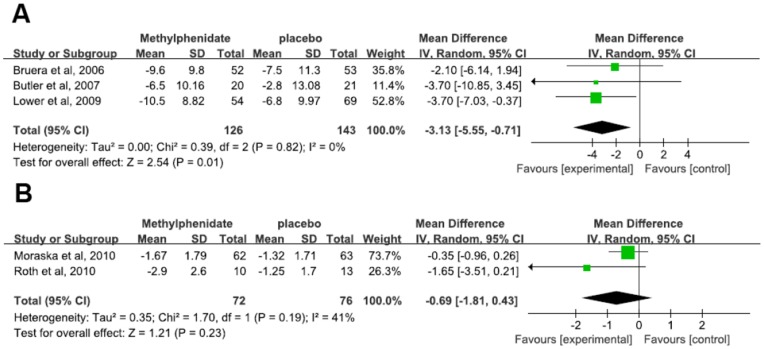
Effects of Methylphenidate on Cancer-Related Fatigue measured by the FACT-F (A) and the BFI (B).

Five RCTs were included to investigate the effect of methylphenidate in the treatment of CRF, consisting of 3 studies of mixed tumor, 1 of primary brain tumor and 1 of prostate cancer with heterogeneous outcome. Three studies measuring fatigue scores used the FACT-F [28]–[29], [31]. In the study of Butler et al., patients underwent a final evaluation at 12 weeks after the completion of treatment. However, there was a high dropout rate over time, and the final analysis was conducted on a smaller sample size than the original one. Despite the fluctuation in fatigue scores at various time points, fatigue scores between methylphenidate and placebo groups were not significantly different at any time points. Hence, we extracted the data at 4 weeks for meta-analysis. The overall mean difference was −3.13 (95% confidence interval [CI] −5.55– −0.71), suggesting significant effect of methylphenidate on CRF (p = 0.01) (Figure 2A). The Other two studies used the BFI [32]–[33], with an overall mean difference of −0.69 (95% [CI] −1.81–0.43), suggesting no significant effect of methylphenidate on CRF (p = 0.23) (Figure 2B). Owing to small number of trials it was not possible to assess the presence of publication bias for each measure of fatigue.

**Figure 3:**
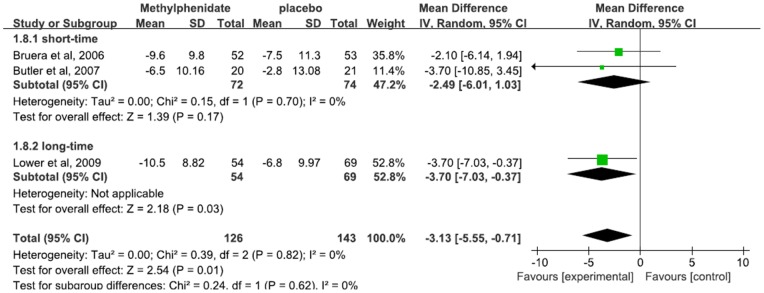
Effects of Methylphenidate with different treatment duration on Cancer-Related Fatigue.

Of note, the studies varied widely in terms of the treatment duration (Bruera et al. 1 week, Butler et al. 4 weeks, Lower et al. 8 weeks). Subgroup analysis was further conducted based on treatment duration, which was dichotomously divided into the long-time (not more than 4 weeks) and short-time group (more than 4 weeks) (Figure 3). Long-time group's overall mean difference was −3.70 (95% [CI] −7.03–−0.37; P = 0.03), and short-time group's overall mean difference was −2.49 (95% [CI] −6.01–1.03; P = 0.17). Compared to treatment with short time duration, long-time treatment with methylphenidate demonstrated a superior effect over placebo.

### Depression and Cognition

**Figure 4:**
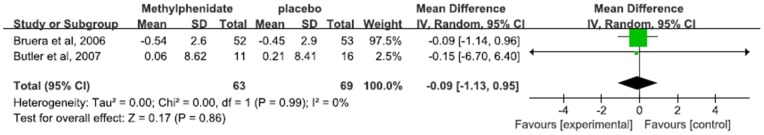
Effect of Methylphenidate on depression associated with CRF.

2 RCTs examined the effect of methylphenidate on depression associated with CRF with consistent outcomes, in which Edmonton Symptom Assessment System-Depression subscale (ESAS-D) was used in 1 study and Center of Epidemiological Study-Depression Scale (CES-D) in another. The pooled standardized mean difference demonstrated no impact of methylphenidate on depression associated with CRF (−0.09, 95%CI −1.13–0.95, p = 0.86, I^2^ = 0%) (Figure 4).

**Figure 5:**
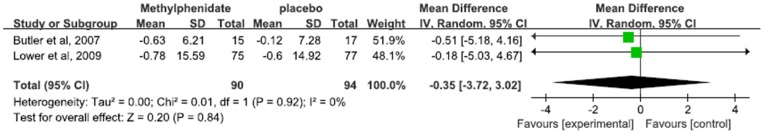
Effect of Methylphenidate on cognition associated with CRF.

The effect of methylphenidate on cognition was examined in 2 RCTs, one used Mini-Mental State Exam (MMSE) and the other one used High Sensitivity Cognitive Screen (HSCS). The pooled standardized mean difference demonstrated no impact of methylphenidate on cognition associated with CRF (−0.35, 95%CI −3.72–3.02, p = 0.84, I^2^ = 0%) (Figure 5).

### Adverse effects

**Figure 6:**
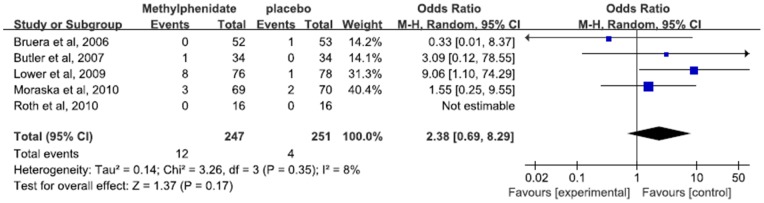
Forest plot shows the incidence of adverse effects in patients assigned to Methylphenidate versus placebo.

Of 5 studies included, the adverse effects were described in 4.9% of patients in methylphenidate group and 1.6% of patients in placebo group. The overall risk ratio for study discontinuation due to side effects didn't suggest statistical significance between patients treated with methylphenidate and patients with placebo (RR 2.38, 95% CI 0.69–8.29, p = 0.17, I^2^ = 8%) (Figure 6).

Occurrence of adverse events reported in the included studies was summarized in Table 2. Totally, more patients reported vertigo, anxiety, anorexia and nausea in methylphenidate group compared to placebo group. Other rates of adverse events were similar between the two groups.

**Table 2:** The pooled adverse effects of Methylphenidate in included studies.

Adverse effect	No. of studies	No. of patients Methylphenidate/placebo	Risk Ratio and 95%CI	P value	I^2^ (%)
Tachycardia	3	96/100	0.66 [0.21, 2.06]	0.47	40
Insomnia	2	128/131	1.46 [0.78, 2.74]	0.23	0
Vertigo	2	128/131	3.74 [1.52, 9.16]	0.004	0
Anxiety	2	145/148	2.50 [1.32, 4.73]	0.005	0
Nausea	2	110/112	4.65 [1.84, 11.77]	0.001	0
Anorexia	2	121/123	2.18 [1.15, 4.14]	0.02	0

## Discussion

Our primary research question was aimed at assessing the effects of methylphenidate on CRF and its safety. This review identified 5 RCTs concerning 498 patients with different types of tumor were enrolled. Despite a large placebo effect observed in the studies included, pooled data suggested therapeutic effect of methylphenidate on CRF and the efficacy of methylphenidate on CRF is getting better with prolonging treatment duration. There was no impact of methylphenidate on depression and cognition associated with CRF. The analysis of serious adverse effects failed to demonstrate any significant differences between groups, except that more patients reported vertigo, anxiety, anorexia and nausea in methylphenidate group compared to placebo group.

Fatigue is a frequent complaint among patients with cancer. Currently fatigue is identified by the response to a single item on a more general health questionnaire or from one or two symptom criteria from symptom checklists [40]. FACT-F is a multidimensional fatigue scale and BFI is a multi-item (unidimensional) measure of CRF. They are both employed in the studies of CRF. In order to avoid introduction of possible heterogeneity into the results, we didn't standardize data with different measure tools. The meta-analysis of fatigue examined by FACT-F showed beneficial effect of methylphenidate. Otherwise, no significant effect of methylphenidate was seen in the meta-analysis of fatigue determined by BFI. FACT-F is used to assess both fatigue and its consequences in patients with a variety of cancers receiving various treatments, which is sensitive to change over time [37]. BFI was developed for screening and assessing clinical outcomes in severely fatigued patients with cancer, with limitation to severity assessment [38]. The different profiles of two scales might contribute to the inconsistency between the two meta-analyses. Moreover, moderate heterogeneity (I^2^ = 41%) and extremely unbalanced weight of the two studies were observed in the pooled data of BFI, which might be one of the potential explanations why therapeutic effect of methylphenidate was not confirmed in the meta-analyses of BFI. Our data were in accordance with the study of Minton et al., in which they drew their conclusion based on studies with a standardized mean difference of FACT-F and BFI [26]. In our study, the result should be interpreted with caution due to a limited number of participants and unbalanced weight of the studies.

Subgroup analysis was conducted based on treatment duration. Compared to treatment with short time duration, long-time treatment demonstrated a superior effect over placebo. It suggested that treatment duration may influence the efficacy of methylphenidate on CRF. Of note, the trial of Lower et al., which reported benefit for methylphenidate, differed from other trials in several ways [31]. The population for other trials included a heterogeneous group of cancers with almost equal numbers of men and women and a slight majority of participants with later-stage disease. The population in the Lower et al. study was almost all female and primarily had breast cancer, with a few women having ovarian cancer. Stage of disease in the Lower et al sample is not specified. In recent trials, they attempted to determine whether any groups of participants found methylphenidate to be helpful [34]. Hence, the interpretation of subgroup analysis should be with caution. Apart from treatment duration, there is variation in clinical characteristics among different trials, which makes interpretation more difficult. In the study of Moraska et al., the overall results didn't show any statistically significant benefit for methylphenidate compared with placebo for alleviating cancer-related fatigue [32]. However, a subset analysis demonstrated that patients with more severe fatigue and/or with more advanced disease did have some fatigue improvement with methylphenidate. It will be probably useful to include patients with more severe fatigue and/or with more advanced diseases in the future trials to avoid a confounder.

Depression is one of the most prevalent comorbids with cancers and patients with depression frequently present fatigue [41]. In the existing studies, depression was well described. Although we found there was no impact of methylphenidate on depression and cognition associated with CRF, we could not evaluate the confounding effect of depression on the effect of methylphenidate. Due to the fact that depression may be one of the causes responsible for CRF, it will be probably useful to exclude patients with depression from RCT in the future to avoid a confounder.

The analysis of serious adverse effects failed to demonstrate any significant differences between groups except that more patients reported vertigo, anxiety, anorexia and nausea in methylphenidate group compared to placebo group. This finding is supported by the results of a recent review of safety concerns regarding the long-term use of methylphenidate [42]. The author of this review identified 26 trials and concluded that adverse effects were minimal in short-term use. There are no data available on the long-term use of these drugs in any condition other than ADD. Although methylphenidate has been used in pediatric cancer survivors for cognitive deficits [43], these drugs cannot be recommended for long-term use in adult cancer survivors as it is likely that the potential benefits are more than outweighed by the concerns over long-term adverse effects.

There are several limitations in our study. Firstly, only a few data from RCTs are available although there is a quantity of case reports and uncontrolled trials. It has to be aware that many of the included studies involved only a small number of participants and failed to follow a consistent research methodology. Further, fatigue is a subjective symptom and can only be assessed subjectively by definition. Due to the diversity of subjective tools, different instruments had better be used simultaneously to assess fatigue thoroughly. Moreover, the mechanisms of fatigue remain poorly defined, which seem to be multifactorial resulting from primary diseases related and other secondary factors. Apart from primary diseases, patients with CRF can suffer concomitant conditions e.g. anxiety, depression and sleep disorders, which can attribute to fatigue as well. In the current studies, patients were not well defined for their psychiatric conditions which could be a confounding factor in the interpretation of trials. It will be probably helpful to exclude patients with sleep disorders and psychiatric conditions from methylphenidate-RCT in the future to avoid a confounder. Finally, methylphenidate in the identified studies was administrated in the short-term treatment. Despite no severe adverse events were presented in the current research, the safety of methylphenidate in the long-term administration, especially abuse or addictive potential, need to be investigated in the future trials. Considering limitations above and there are additional studies ongoing, the evidence about the use of methylphenidate in CRF is likely to continue to evolve.

## Conclusions

Our pooling data support the viewpoint that methylphenidate may be effective in the management of CRF. However, all studies had small sample sizes. In the absence of convincing results from a single, large, well-conducted randomized controlled trial, this advice must be considered to be tentative and provisional. If methylphenidate were to be used in patients with CRF, it should only be prescribed under expert supervision and with active monitoring. Nonetheless, methylphenidate is one of the few interventions available to treat CRF that are supported by trial data. Further research is needed before their use can be recommended more widely.

## Supporting Information

Checklist S1 PRISMA checklist.
(DOC)
